# Factors predicting relapse and treatment discontinuation with paliperidone 3-monthly long-acting injection: A 2-year naturalistic follow-up study

**DOI:** 10.1192/j.eurpsy.2021.2243

**Published:** 2021-10-26

**Authors:** Ivana Clark, Phoebe Wallman, Victoria Cornelius, David Taylor

**Affiliations:** 1 Pharmacy Department, South London and Maudsley NHS Foundation Trust, Denmark Hill, London SE5 8AZ, UK; 2 School of Public Health, Imperial College London, Stadium House, 68 Wood Lane, London W12 7RH, UK; 3 Institute of Pharmaceutical Science, King’s College, 5th Floor, Franklin-Wilkins Building, 150 Stamford Street, London SE1 9NH, UK

**Keywords:** discontinuation, paliperidone, psychotic disorders, relapse, three monthly

## Abstract

**Background:**

Paliperidone 3-monthly (PP3M) long-acting injection has proven efficacy and effectiveness in schizophrenia. Little is known of its effectiveness in other diagnoses.

**Methods:**

All patients starting PP3M were followed up for 2 years. Main outcome measures were relapse and discontinuation from PP3M. *Post hoc* we examined outcomes in those switched back to one monthly paliperidone (PP1M) long-acting injection.

**Results:**

Overall, 186 patients were followed-up. At the 2-year end point, 110 patients (59%) were still receiving PP3M, and 129 (70%) were receiving some form of paliperidone long-acting injection. Discontinuation from paliperidone long-acting injections (PPLAIs) was more likely with a nonschizophrenia diagnosis (hazard ratio [HR] for continuation 0.429 [95% confidence intervals (CI) – 0.21, 0.87 *p* = 0.018)), and prior clozapine use [in PP3M patients; HR for discontinuation 1.87 [95% CI – 1.05, 3.30 *p* = 0.032]). Relapse occurred in 20 (11%) of those receiving PP3M. Relapse on PP3M and PPLAIs was more likely in nonschizophrenia diagnosis (HR 0.17 for remaining relapse-free [95% CI – 0.06, 0.50; *p* = 0.001]; HR 0.21 [95% CI – 0.08, 0.58 *p* = 0.002], respectively), polypharmacy in PP3M patients (HR for relapse 7.91 [95% CI – 3.73, 22.9; *p* < 0.001]) and PPLAI patients (HR for relapse 6.45 [95% CI – 2.49, 16.5; *p* < 0.001]), and prior clozapine use in PP3M patients (HR for relapse 6.11 [95% CI – 1.82, 20.5; *p* = 0.003]) and PPLAI patients (HR for relapse 4.52 (95% CI – 1.51, 13.5; *p* = 0.007).

**Conclusions:**

Outcomes with PP3M are excellent in practice, even when used outside its formal license. PP3M was relatively more effective in those with an F20 schizophrenia diagnosis and in those never before considered for or prescribed clozapine.

## Introduction

Paliperidone long-acting injections (PPLAIs) are effective in maintenance treatment of adults with schizophrenia and licensed for this use in most of the world. There are relatively limited reports of one monthly paliperidone (PP1M) use in schizoaffective disorder [1–3] or bipolar disorder [3–5]. These studies do however support their value in reducing hospital admissions and the number of manic or mixed episodes, and the risk of relapse and hospitalization. To the best of our knowledge, no studies have examined Paliperidone 3-monthly (PP3M) use in conditions outside schizophrenia.

The main aim of this study was to investigate naturalistic outcomes—relapse and discontinuation—resulting from licensed and unlicensed prescribing of PP3M in clinical practice. A secondary aim was to determine factors relating to relapse and discontinuation.

## Method

We followed up patients treated at the South London and Maudsley NHS foundation trust—a specialist mental health organization that serves 1.2 million people in south east London. The study was approved by the South London and Maudsley Drugs and Therapeutics Committee in 2016 (approval code SLaM/DTC/2016/3) as a service development not requiring individual patient consent. This was a prospective, noninterventional evaluation which did not alter patient treatment in any way. Patients were prescribed PP3M according to clinical need and did not undergo any additional investigation or monitoring. Patient data were collected using the Trust’s bespoke electronic patient record system—ePJS. Patient data were viewed only by pharmacy staff normally involved in patient care and data were anonymized at source and held on a secure database. No patient can be identified in this report.

Our *a priori* main outcomes were continuation with, and relapse on, PP3M in all patients prescribed PP3M regardless of indication. Our secondary aim was to find predictors for these outcomes based on a robust statistical model of the results. All patients starting on PP3M with South London and Maudsley were registered (by prescriber initiation form) and followed up for 2 years from the date of initiation. All included patients received at least one dose of PP3M. The prescriber initiation form included the following information: patient’s age, primary diagnosis (ICD-10), confirmation patient was neither pregnant nor breastfeeding, renal function, and PP1M maintenance doses. Each form was screened by a pharmacist for completeness and kept secure at the pharmacy. Information provided from initiation forms was checked against information stored on the patient electronic record system. Information collected and confirmed included: care setting on initiation, having ever been prescribed or considered for clozapine (a proxy measure for treatment responsiveness/resistance), any additional antipsychotic prescribed over follow-up (excluding PRN) and their PP1M initiation date and dose. Electronic patient medical records were accessed to gain information on outcome (continuation and relapse), reasons for discontinuation and medication prescribed after discontinuation. Additionally, information was collected on the average number of injections per year, time between injections and dose strengths. Adverse effects were reported anonymously to Medicines and Healthcare products Regulatory Agency and Janssen-Cilag Ltd. (Buckinghamshire, UK).

Discontinuation was defined as not receiving PP3M for 4 months (the 3-month dose interval + 1 month). The index date was taken as time of first prescription for PP3M and the date of discontinuation was counted as their last injection plus 3 months. Discontinuation from PP3M owing to prescriber decision relating to a worsening of condition was not, for the purposes of this study, defined as a relapse unless it resulted in a step-up in clinical care. Time to discontinuation was calculated from index to discontinuation or to attrition.

Relapse was defined as a step-up in clinical care and included referral to home-treatment team, attendance in Accident and Emergency (ER) services for reasons of psychiatric deterioration resulting in psychiatric admission, or direct admission to a psychiatric hospital. Time to relapse on PP was taken as time from index to the step-up in care. Discontinuation was treated as a censoring variable in continuous analysis of relapse.

*Post hoc* secondary outcomes were continuation and relapse on all PPLAI formulations (PP3M alone or PP3M followed immediately by PP1M) prescribed over the 2-year follow-up period. Discontinuation from PP1M (post PP3M) was dated as the last injection plus 1 month.

### Statistical analysis

Baseline characteristics were summarized using measures of central tendency and variability. The association between diagnosis and time to relapse and time to discontinuation on PP3M and then PPLAI was examined with use of Cox proportional hazard models.

All regression models were adjusted for age, inpatient (Y/N), sex, polypharmacy, ethnicity, and dose, prior use of clozapine. Model assumptions were examined using Scholfeld’s residuals and plots for each variable in the model and overall. Adjusted cumulative hazard plots by F20 diagnosis were constructed at the mean of model covariates plots. Statistical analysis was conducted in Stata version 15.0 (StataCorp LLC, College Station, TX) and R version 4.0.2. (R Foundation for Statistical Computing, Vienna, Austria).

## Results

In total, 186 patients were initiated on PP3M between 2016 and January 2019.

### Continuation

**Figure 1.**
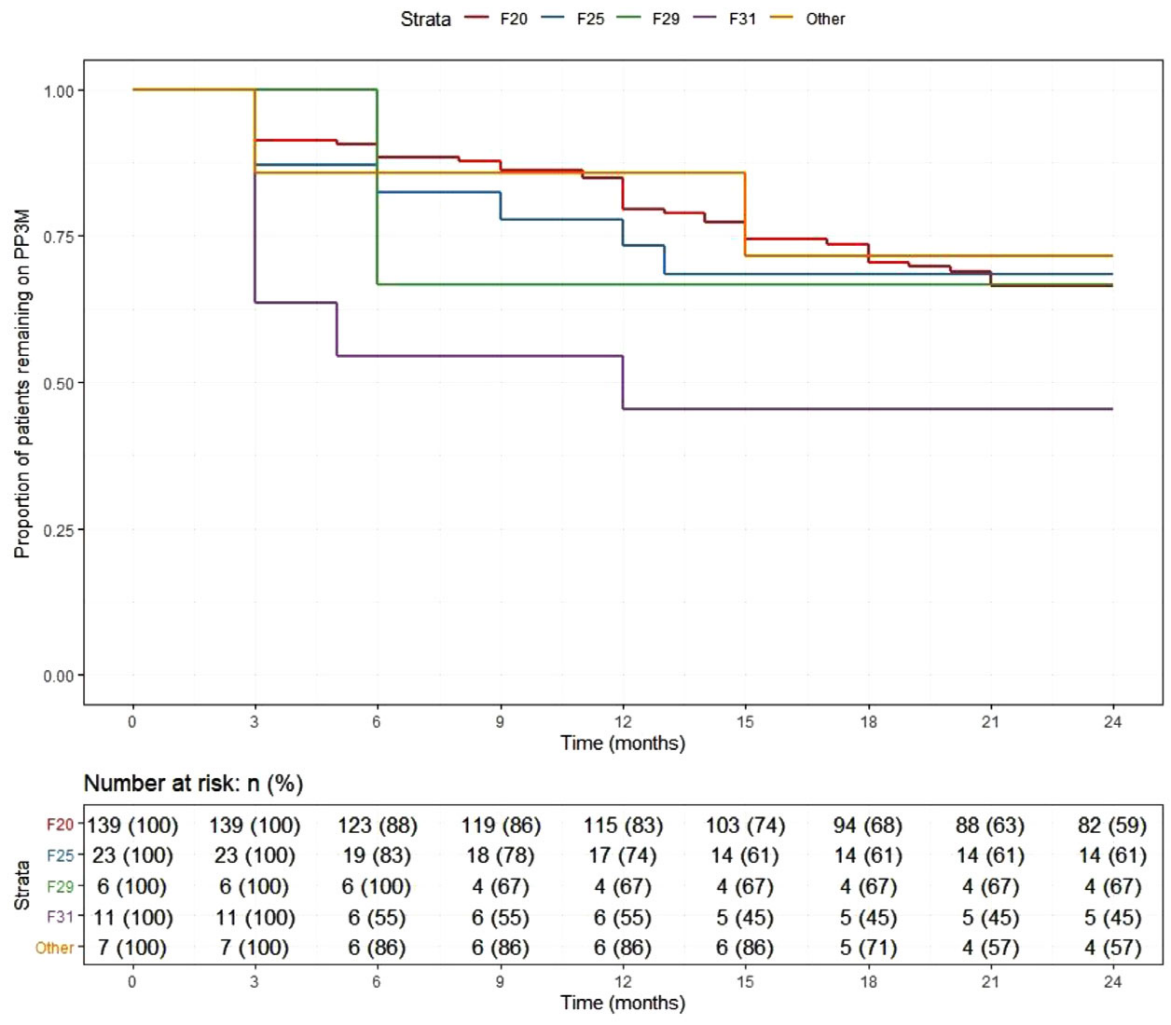
Kaplan–Meier plot showing the proportion of patients prescribed paliperidone 3-monthly (PP3M) since initiation in diagnosis groups.

**Figure 2.**
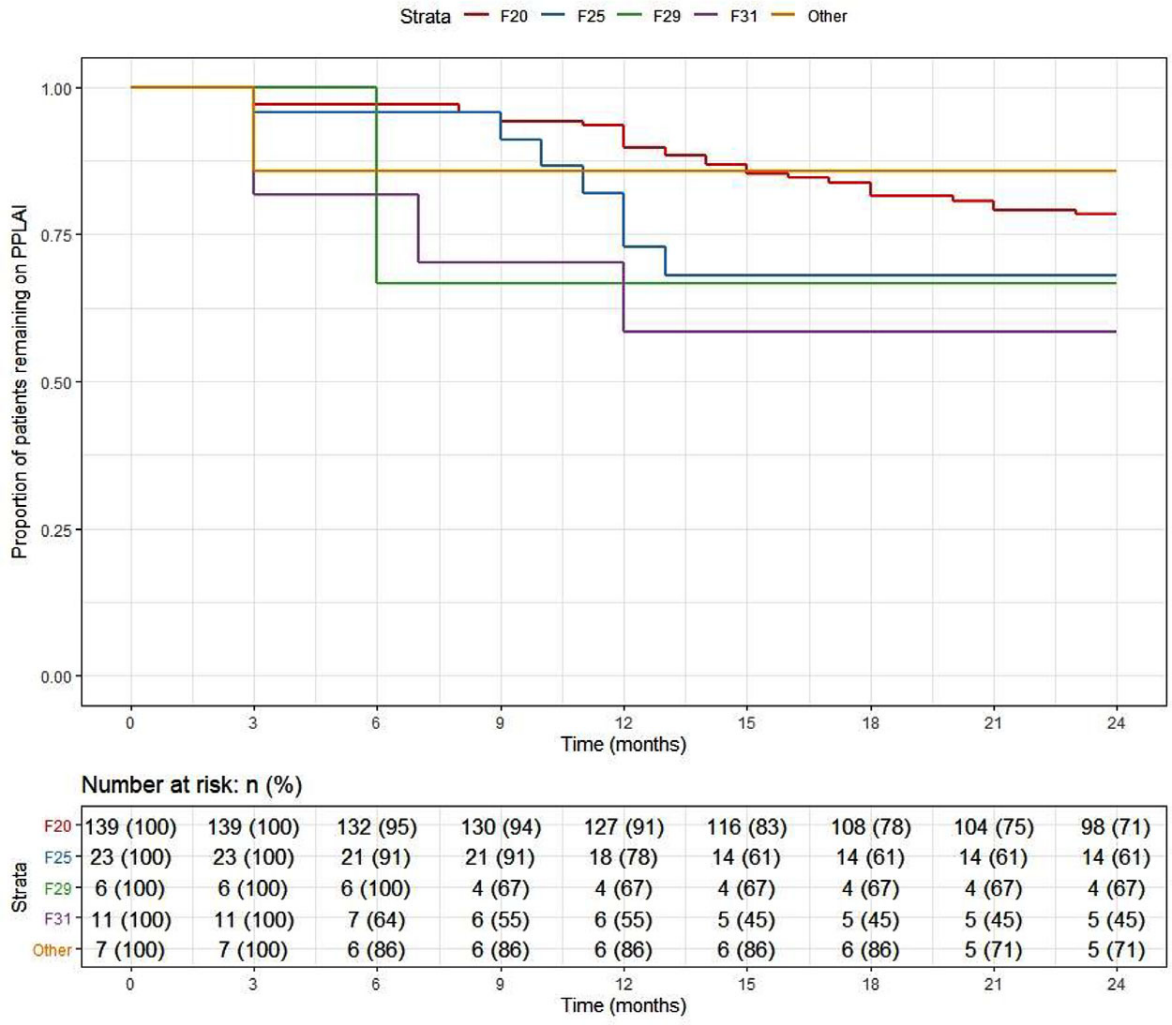
Kaplan–Meier plot showing the proportion of patients prescribed paliperidone long-acting injection (PPLAI) since initiation in diagnosis groups.

At the end of the 2-year follow-up, 110 patients (59%) had continuously received PP3M for the whole study period. Of those who discontinued, nearly half (*n* = 29) changed their medication to PP1M (10 later discontinued). The continuation rate for all PPLAI use from the PP3M initiation date was 70% (129/186). Figures 1 and 2 show these continuation rates within diagnosis groups. The most frequent reasons recorded for discontinuation were patient refusal (*n* = 18), perceived inefficacy (*n* = 15), and adverse effects (*n* = 11) (Table 1). Adverse effects cited as the reason for discontinuing were: hyperprolactinemia (*n* = 4), weight gain (*n* = 4), erectile dysfunction (*n* = 2), restlessness (*n* = 1), blurred vision (*n* = 1), anorgasmia (*n* = 1), extrapyramidal side effects (*n* = 1), gynaecomastia (*n* = 1), anhedonia (*n* = 1), lumps at injection sites (*n* = 1), and deranged liver function test (*n* = 1). Discontinuation statistics are displayed in Table 1. The baseline characteristics for each outcome group are shown in Table 2 and PP3M and PPLAI characteristics in Table 3.

**Table 1.** Discontinuation from PP3M.

Clinical outcome after 2 years (PP3M)		
	*n* = 186	
Outcome 2 years after initiating PP3M [n (%)]		
Continuation	110	(59.1)
Discontinuation	62	(33.3)
Attrition	14	(7.5)
	*n* = 62	
Reasons for discontinuation [*n* (%)]		
Patient refusal	18	(29.0)
Perceived inefficacy	15	(24.2)
Adverse effects	11	(17.7)
Patient request	6	(9.7)
Need more flexible dose adjustment	4	(6.5)
Independent health condition	3	(4.8)
Discharged from mental health services	3	(4.8)
Age concern (>65 years old)	2	(3.2)
Next medication (3 months after last depot) [*n* (%)]		
PP1M	29	(46.8)
No medication	21	(33.9)
Risperidone (oral)	5	(8.1)
Aripiprazole (LAI)	2	(3.2)
Aripiprazole (oral)	1	(1.6)
Clozapine (oral)	1	(1.6)
Flupenthixol (LAI)	1	(1.6)
Haloperidol (LAI)	1	(1.6)
Haloperidol (oral)	1	(1.6)
	*n* = 14	
*Reasons for attrition* [*n* (%)]		
Lost to follow-up^a^	8	(57.1)
Died^b^	6	(42.9)

Abbreviations: LAI, long-acting injection; PP3M, Paliperidone 3-monthly.

a Lost to follow up: left country (*n* = 4), missing person (*n* = 2), changed trust (*n* = 1), and disengagement (*n* = 1).

b Deaths classified using ePJS and not at a systems level: unknown (*n* = 2), heroin overdose (*n* = 1), natural causes (*n* = 1), nonadherence to diabetes medication (*n* = 1), and sepsis (*n* = 1).

**Table 2.** Baseline characteristics for continuation outcomes on PP3M and PPLAI.

Characteristic	Total		PP3M continuers		PP3M discontinuers^a^		PPLAI continuers		PPLAI discontinuers^a^	
	*n* = 186		*n* = 110		*n* = 76		*n* = 129		*n* = 57	
Age on initiation of PP3M (years)										
Mean, SD	45, 13.2		46, 11.7		44, 15.1		47, 12.3		42, 14.4	
Min, max	20, 77		22, 74		20, 77		22, 77		20, 74	
Prior duration of PP1M (months)										
Mean, SD	34, 24.5		35, 25.1		34, 23.7		37, 24.9		28, 22.5	
Min, max	3, 89		3, 89		3, 81		3, 89		3, 81	

	*n* (%)		*n* (%)		*n* (%)		*n* (%)		*n* (%)	
Within licensed age range										
No	15	(8.1)	8	(7.3)	7	(9.2)	11	(8.5)	4	(7.0)
Yes	171	(91.9)	102	(92.7)	69	(90.8)	118	(91.5)	53	(93.0)
Female	62	(33.3)	32	(29.1)	30	(39.5)	43	(33.3)	19	(33.3)
Male	124	(66.7)	78	(70.9)	46	(60.5)	86	(66.7)	38	(66.7)
Asian/Asian British^b^	11	(5.9)	8	(7.3)	3	(3.9)	8	(6.2)	3	(5.3)
Black/Black British	107	(57.5)	64	(58.2)	43	(56.6)	75	(58.1)	32	(56.1)
Mixed background	9	(4.8)	4	(3.6)	5	(6.6)	4	(3.1)	5	(8.8)
Other	14	(7.5)	7	(6.4)	7	(9.2)	8	(6.2)	6	(10.5)
White	45	(24.2)	27	(24.5)	18	(23.7)	34	(26.4)	11	(19.3)
Diagnosis										
F10	1	(0.5)	0	(0.0)	1	(1.3)	1	(0.8)	0	(0.0)
F11	1	(0.5)	0	(0.0)	1	(1.3)	0	(0.0)	1	(1.8)
F20	139	(74.7)	83	(75.5)	56	(73.7)	99	(76.7)	40	(70.2)
F22	3	(1.6)	3	(2.7)	0	(0.0)	3	(2.3)	0	(0.0)
F23	1	(0.5)	0	(0.0)	1	(1.3)	0	(0.0)	1	(1.8)
F25	23	(12.4)	14	(12.7)	9	(11.8)	14	(10.9)	9	(15.8)
F29	6	(3.2)	4	(3.6)	2	(2.6)	4	(3.1)	2	(3.5)
F31	11	(5.9)	5	(4.5)	6	(7.9)	7	(5.4)	4	(7.0)
F32	1	(0.5)	1	(0.9)	0	(0.0)	1	(0.8)	0	(0.0)
Care setting on initiation of PPLAI										
Inpatient	104	(55.9)	57	(51.8)	47	(61.8)	67	(51.9)	37	(64.9)
Outpatient	82	(44.1)	53	(48.2)	29	(38.2)	62	(48.1)	20	(35.1)
Considered treatment-resistant										
No	135	(72.6)	86	(78.2)	49	(64.5)	95	(73.6)	40	(70.2)
Yes	51	(27.4)	24	(21.8)	27	(35.5)	34	(26.4)	17	(29.8)
Prior PP1M dose										
150 mg	70	(37.6)	39	(35.5)	31	(40.8)	46	(35.7)	24	(42.1)
100 mg	68	(36.5)	37	(33.6)	31	(40.8)	43	(33.3)	25	(43.9)
75 mg	30	(16.1)	20	(18.2)	10	(13.2)	24	(18.6)	6	(10.5)
50 mg	18	(9.7)	14	(12.7)	4	(5.3)	16	(12.4)	2	(3.5)

Abbreviations: PPLAI, paliperidone long-acting injection; PP1M, one monthly paliperidone; PP3M, Paliperidone 3-monthly; SD, standard deviation.

a Discontinuers includes those who stopped due to death or being lost to follow-up.

b Asian refers to Indian—subcontinent as per NHS ethnicity classification.

**Table 3.** PP3M and PPLAI characteristics for continuation.

PP3M	Total		PP3M continuers		PP3M discontinuers^a^		PPLAI continuers		PPLAI discontinuers^a^	
	*n* = 186		*n* = 110		*n* = 76		*n* = 129		*n* = 57	
Average PP3M injections/year =										
Mean, SD	4.0, 0.1		4.0, 0.1		4.0, 0.2		4.0, 0.1		4.0, 0.2	
Min, max	3.6, 4.7		3.6, 4.6		3.6, 4.7		3.6, 4.6		3.6, 4.7	
Average no. days between PP3M injections										
Mean, SD	92, 3.5		92, 2.6		92, 5.0		93, 2.6		92, 5.5	
Min, max	70, 106		86, 105		70, 106		86, 105		70, 106	

	*n* (%)		*n* (%)		*n* (%)		*n* (%)		*n* (%)	
Initial PP3M dose										
525 mg	64	(34.4	38	(34.5)	26	(34.2)	43	(33.3)	21	(36.8)
350 mg	71	(38.2)	38	(34.5)	33	(43.4)	45	(34.9)	26	(45.6)
263 mg	32	(17.2)	20	(18.2)	12	(15.8)	25	(19.4)	7	(12.3)
175 mg	19	(10.2)	14	(12.7)	5	(6.6)	16	(12.4)	3	(5.3)
Dose change										
None	173	(93.0)	101	(91.8)	72	(94.7)	118	(91.5)	55	(96.5)
Increase	3	(1.6)	1	(0.9)	2	(2.6)	3	(2.3)	0	(0.0)
Worsening symptoms	3	(100)	1	(100)	2	(100)	3	(100)	0	(0.0)
Decrease	10	(5.4)	8	(7.3)	2	(2.6)	8	(6.2)	2	(3.5)
Adverse effects	5	(50.0)	3	(37.5)	2	(100)	3	(37.5)	2	(100)
Patient request	3	(30.0)	3	(37.5	0	(0.0)	3	(37.5	0	(0.0)
Improved condition	1	(10.0)	1	(12.5)	0	(0.0)	1	(12.5)	0	(0.0)
Unclear	1	(10.0)	1	(12.5)	0	(0.0)	1	(12.5)	0	(0.0)
Polypharmacy										
No	158	(84.9)	95	(86.4)	63	(82.9)	111	(86.0)	47	(82.5)
Yes	28	(15.1)	15	(13.6)	13	(17.1)	18	(14.0)	10	(17.5)

Abbreviations: PPLAI, Paliperidone 3-monthly; PP3M, paliperidone long-acting injection; SD, standard deviation.

a Discontinuers includes those who stopped due to death or being lost to follow-up.

### Relapse

**Figure 3.**
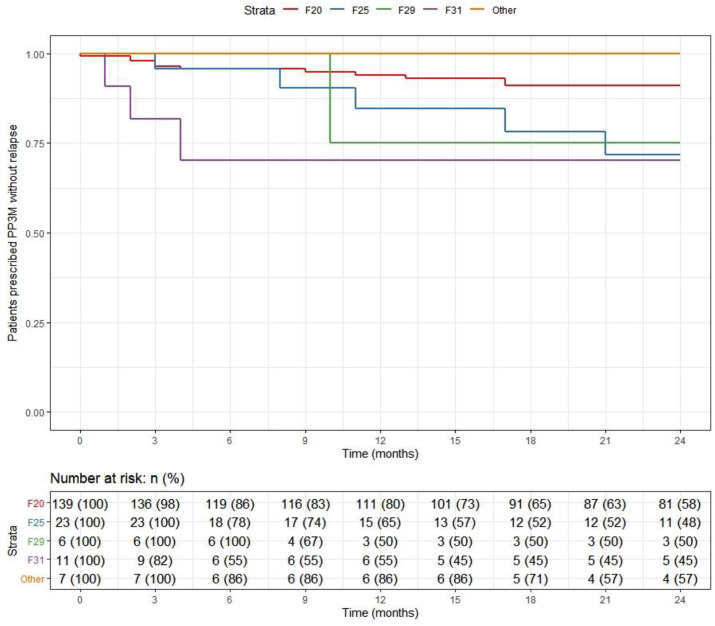
Kaplan–Meier plot showing the proportion of patients that relapsed while being prescribed Paliperidone 3-monthly (PP3M) since initiation in diagnosis groups.

**Figure 4.**
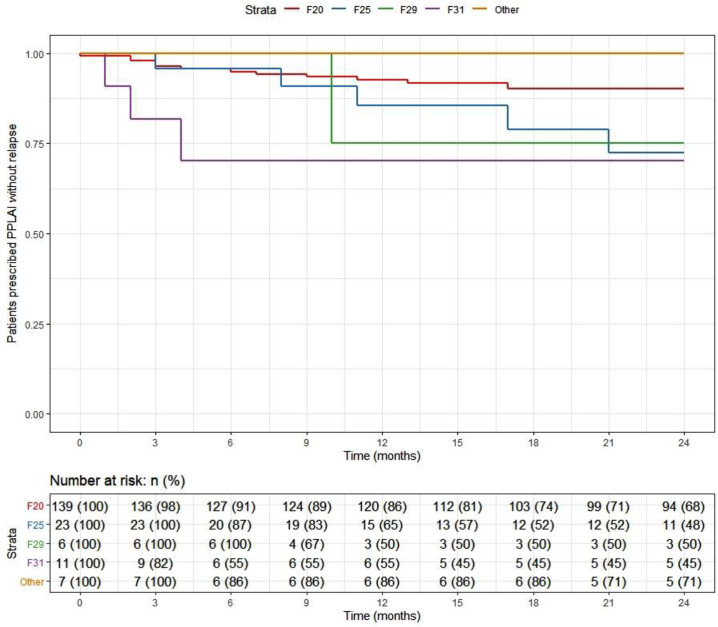
Kaplan–Meier plot showing the proportion of patients that relapsed while being prescribed paliperidone long-acting injection (PPLAI) since initiation in diagnosis groups.

Overall, 166 patients (89%) who began treatment on PP3M remained relapse-free over the 2-year follow-up. The relapse-free rate was 88% (164/186) patients in the cohort on PPLAI. Figures 3 and 4 show these relapse rates within diagnosis groups. Tables 4 and 5 show the baseline and PP characteristics for those who relapsed and those who did not.

**Table 4.** Baseline characteristics for relapse outcomes on PP3M and PPLAI.

Characteristic	Total		PP3M no relapse		PP3M relapse		PPLAI no relapse		PPLAI relapse	
	*n* = 186		*n* = 166		*n* = 20		*n* = 164		*n* = 22	
Age on initiation of PP3M (years)										
Mean, SD	45, 13.2		46, 12.8		40, 15.9		46, 12.8		40, 15.3	
Min, max	20, 77		20, 77		22, 77		20, 77		22, 77	
Prior duration of PP1M (months)										
Mean, SD	34, 24.5		34, 24.6		33, 24.1		34, 24.6		35, 23.8	
Min, max	3, 89		3, 89		4, 75		3, 89		4, 75	

	*n* (%)		*n* (%)		*n* (%)		*n* (%)		*n* (%)	
Within licensed age range										
No	15	(8.1)	13	(7.8)	2	(10.0)	13	(7.9)	2	(9.1)
Yes	171	(91.9)	153	(92.2)	18	(90.0)	151	(92.1)	20	(90.9)
Sex										
Female	62	(33.3)	54	(32.5)	8	(40.0)	53	(32.3)	9	(40.9)
Male	124	(66.7)	112	(67.5)	12	(60.0)	111	(67.7)	13	(59.9)
Ethnicity										
Asian/Asian British^a^	11	(5.9)	9	(5.4)	2	(10.0)	9	(5.5)	2	(9.1)
Black/Black British	107	(57.5)	97	(58.4)	10	(50.0)	95	(57.9)	12	(54.5)
Mixed background	9	(4.8)	9	(5.4)	0	(0.0)	9	(5.5)	0	(0.0)
Other	14	(7.5)	11	(6.6)	3	(15.0)	11	(6.7)	3	(13.6)
White	45	(24.2)	40	(24.1)	5	(25.0)	40	(24.4)	5	(22.7)
Diagnosis										
F10	1	(0.5)	1	(0.6)	0	(0.0)	1	(0.6)	0	(0.0)
F11	1	(0.5)	1	(0.6)	0	(0.0)	1	(0.6)	0	(0.0)
F20	139	(74.7)	128	(77.1)	11	(55.0)	126	(76.8)	13	(59.1)
F22	3	(1.6)	3	(1.8)	0	(0.0)	3	(1.8)	0	(0.0)
F23	1	(0.5)	1	(0.6)	0	(0.0)	1	(0.6)	0	(0.0)
F25	23	(12.4)	18	(10.8)	5	(25.0)	18	(11.0)	5	(22.7)
F29	6	(3.2)	5	(3.0)	1	(5.0)	5	(3.0)	1	(4.5)
F31	11	(5.9)	8	(4.8)	3	(15.0)	8	(4.9)	3	(13.6)
F32	1	(0.5)	1	(0.6)	0	(0.0)	1	(0.6)	0	(0.0)
Care setting on initiation of PPLAI										
Inpatient	104	(55.9)	93	(56.0)	11	(55.0)	91	(55.5)	13	(59.1)
Outpatient	82	(44.1)	73	(44.0)	9	(45.0)	73	(44.5)	9	(40.9
Considered treatment-resistant										
No	135	(72.6)	124	(74.7)	11	(55.0)	123	(75.0)	12	(54.5)
Yes	51	(27.4)	42	(25.3)	9	(45.0)	41	(25.0)	10	(45.5)
Prior PP1M dose										
150 mg	70	(37.6)	57	(34.3)	13	(65.0)	56	(34.1)	14	(63.6)
100 mg	68	(36.5)	64	(38.6)	4	(20.0)	63	(38.4)	5	(22.7)
75 mg	30	(16.1)	29	(17.5)	1	(5.0)	29	(17.7)	1	(4.5)
50 mg	18	(9.7)	16	(9.6)	2	(10.0)	16	(9.8)	2	(9.1)

Abbreviations: PPLAI, paliperidone long-acting injection; PP1M, one monthly paliperidone; PP3M, Paliperidone 3-monthly; SD, standard deviation.

a Discontinuers includes those who stopped due to death or being lost to follow-up.

**Table 5.** PP3M characteristics for relapse outcomes on PP3M and PPLAI.

PP3M	Total		PP3M no relapse		PP3M relapse		PPLAI no relapse		PPLAI relapse	
	*n* = 186		*n* = 166		*n* = 20		*n* = 164		*n* = 22	
Average PP3M injections/year										
Mean, SD	4.0, 0.1		4.0, 0.1		4.0, 0.1		4.0, 0.1		4.0, 0.1	
Min, max	3.6, 4.7		3.6, 4.7		3.7, 4.2		3.6, 4.7		3.7, 4.2	
Average no. days between PP3M injections										
Mean, SD	92, 3.5		92, 3.4		93, 4.6		92, 3.4		92, 4.4	
Min, max	70, 106		70, 106		84, 105		70, 106		84, 105	

	*n* (%)		*n* (%)		*n* (%)		*n* (%)		*n* (%)	
Initial PP3M dose										
525 mg	64	(34.4	55	(33.1)	9	(45.0)	54	(32.9)	10	(45.5)
350 mg	71	(38.2)	64	(38.6)	7	(35.0)	64	(39.0)	7	(31.8)
263 mg	32	(17.2)	30	(18.1)	2	(10.0)	29	(17.7)	3	(13.6)
175 mg	19	(10.2)	17	(10.2)	2	(10.0)	17	(10.4)	2	(9.1)
Dose change										
None	173	(93.0)	155	(93.4)	18	(90.0)	153	(93.3)	20	(90.9)
Increase	3	(1.6)	2	(1.2)	1	(5.0)	2	(1.2)	1	(4.5)
Worsening symptoms	3	(100)	2	(100)	1	(100)	2	(100)	1	(100)
Decrease	10	(5.4)	9	(5.4)	1	(5.0)	9	(5.5)	1	(4.5)
Adverse effects	5	(50.0)	5	(55.6)	0	(0.0)	5	(55.6)	0	(0.0)
Patient request	3	(30.0)	2	(22.2)	1	(100)	2	(22.2)	1	(100)
Improved condition	1	(10.0)	1	(11.1)	0	(0.0)	1	(11.1)	0	(0.0)
Unclear	1	(10.0)	1	(11.1)	0	(0.0)	1	(11.1)	0	(0.0)
Polypharmacy										
No	158	(84.9)	148	(89.2)	10	(50.0)	147	(89.6)	11	(50.0)
Yes	28	(15.1)	18	(10.8)	10	(50.0)	17	(10.4)	11	(50.0)

Abbreviations: PPLAI, paliperidone long-acting injection; PP3M, Paliperidone 3-monthly; SD, standard deviation.

**Table 6.** Regression modeling for time to relapse and discontinuation from PP3M and PPLAI.

Model	Time to relapse (PP3M)			Time to discontinuation (PP3M)			Time to relapse (PPLAI)			Time to discontinuation (PPLAI)		
Covariates	HR	95% CI	*p*-value	HR	95% CI	*p*-value	HR	95% CI	*p*-value	HR	95% CI	*p*-value
Age	0.97	(0.93, 1.00)	0.056	0.97	(0.95, 0.99)	0.017	0.96	(0.93, 0.99)	0.045	0.95	(0.93, 0.98)	<0.001
Inpatient	0.81	(0.31, 2.10)	0.662	1.54	(0.89, 2.65)	0.121	0.90	(0.36,2.23)	0.816	1.75	(0.88, 3.47)	0.109
Male gender	0.84	(0.31, 2.31)	0.741	0.52	(0.30, 0.88)	0.016	0.79	(0.32,1.92)	0.606	0.60	(0.31, 1.17)	0.132
Polypharmacy	7.91	(2.73, 22.9)	<0.001	1.05	(0.53, 2.04)	0.897	6.45	(2.49, 16.5)	<0.001	0.92	(0.42, 1.99)	0.831
Ethnicity	1.37	(0.48, 4.21)	0.586	1.18	(0.65, 2.15)	0.581	1.19	(0.41,3.48)	0.754	0.79	(0.35,1.79)	0.572
Dose	0.86	(0.48, 1.51)	0.594	0.97	(0.73, 1.31)	0.857	0.90	(0.52,1.54)	0.692	1.13	(0.78, 1.63)	0.524
Prior clozapine	6.11	(1.82, 20.5)	0.003	1.87	(1.05, 3.30)	0.032	4.52	(1.51, 13.5)	0.007	1.15	(0.54, 2.41)	0.721
F20	0.17	(0.06, 0.50)	0.001	0.62	(0.34, 1.13)	0.119	0.21	(0.08, 0.58)	0.002	0.429	(0.21, 0.87)	0.018

Abbreviations: CI, confidence intervals; HR, hazard ratio; PPLAI, paliperidone long-acting injection; PP3M, Paliperidone 3-monthly; SD, standard deviation.

### Regression analysis

The regression analysis for outcomes is displayed in Table 6. The hazard ratio for relapse while on PP3M and PPLAI was 0.17 and 0.21, respectively for those with an F20 diagnosis compared with those with another diagnosis. Prior clozapine use was associated with a markedly increased risk of relapse 6.11-fold (on PP3M and 4.52 on PPLAI) as was polypharmacy, which increased risk 7.91-fold (on PP3M and 6.45 on PPLAI). For every additional year of patient age the risk of relapse on PPLAI increased by 4%.

Older age was also associated with discontinuation, 3% increased risk per year on PP3M and 5% on PPLAI. Being male and prior clozapine use were associated with discontinuation from PP3M and a non-F20 diagnosis when on PPLAI.

**Figure 5.**
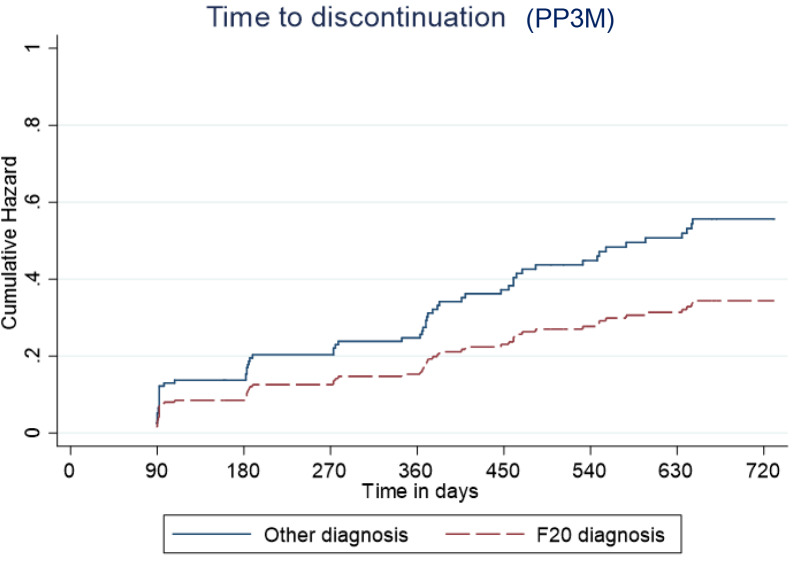
Kaplan–Meier plot showing the predictive cumulative hazard for discontinuation from Paliperidone 3-monthly (PP3M) in days using the regression model. F20 diagnosed patients are represented in red and non-F20 in blue.

**Figure 6.**
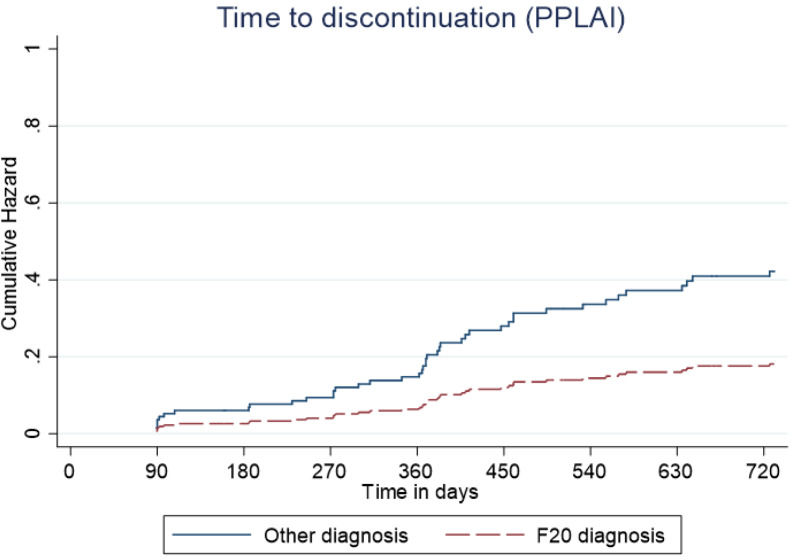
Kaplan–Meier plot showing the predictive cumulative hazard for discontinuation from paliperidone long-acting injection (PPLAI) in days using the regression model. F20 diagnosed patients are represented in red and non-F20 in blue.

**Figure 7.**
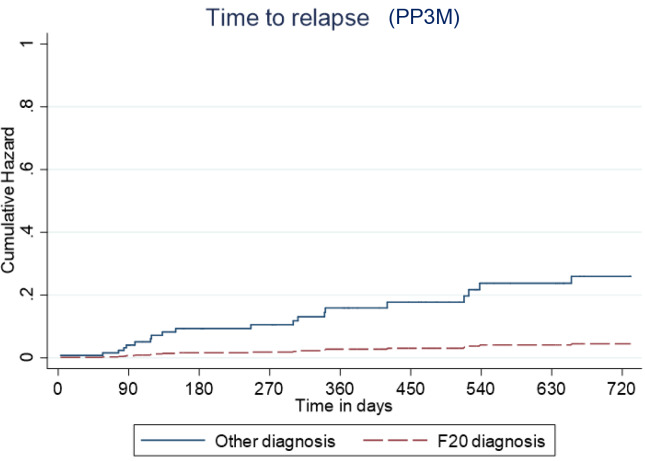
Kaplan–Meier plot showing the predictive cumulative hazard for relapse on Paliperidone 3-monthly (PP3M) in days using the regression model. F20 diagnosed patients are represented in red and non-F20 in blue.

**Figure 8.**
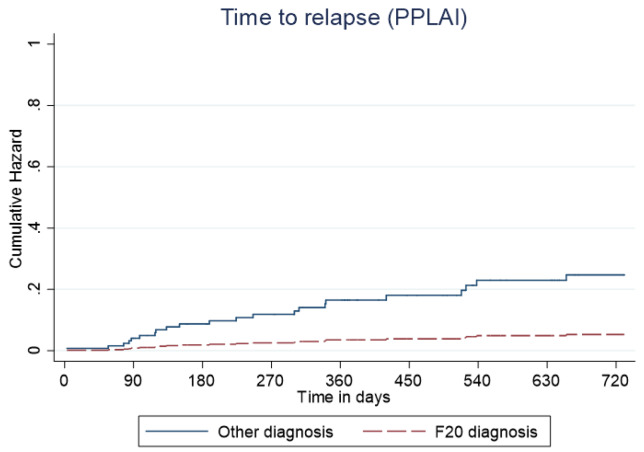
Kaplan–Meier plot showing the predictive cumulative hazard for relapse on paliperidone long-acting injection (PPLAI) in days using the regression model. F20 diagnosed patients are represented in red and non-F20 in blue.

Predictive cumulative and adjusted hazards for the outcome events for those with and an F20 diagnosis and those with another diagnosis are shown in Figures 5–8.

## Discussion

Overall, 70% of patients started on PP3M were still on PPLAI in some form 2 years later, and most were still on PP3M. Relapse over the 2 years after starting PP3M was 11% with PP3M and 12% with any paliperidone formulation. These very positive results were largely driven by the licensed use of PP3M in people with a diagnosis of schizophrenia (F20). We found that patients with an F20 diagnosis had an 83% reduced risk of relapse on PP3M compared with non-F20 patients. An F20 diagnosis was also associated with a lower likelihood of PPLAI discontinuation. In addition to this, prior clozapine use and concurrent polypharmacy were associated with a substantially greater risk of relapse while on PPLAIs. Increased age was also linked to a somewhat higher rate of discontinuation from PPLAIs.

## Discontinuation

The association between age and discontinuation was statistically significant, but the effect was small and probably clinically insignificant. The strong link between polypharmacy and discontinuation is difficult to explain given the observational nature of this study. Polypharmacy regimes are more poorly tolerated than monotherapy and are associated with higher adverse effect burden [6]. Polypharmacy is also often an indication of more difficult-to-treat illness, although in our study nearly half of patients on polypharmacy were co-prescribed aripiprazole. Locally, it is our practice to use aripiprazole to treat or prevent hyperprolactinaemia rather than because of poor response. The association between prior clozapine therapy (our proxy for treatment resistance) and increased risk of discontinuation might have reflected a relatively poor response to paliperidone and subsequent discontinuation. Clozapine is, in our trust, only used as a treatment for refractory schizophrenia so prior use is assumed to indicate poor response to non-clozapine antipsychotics.

The association between non-F20 diagnoses (in this study, mainly schizoaffective and bipolar disorders) with higher discontinuation rates is on one level not surprising as in the UK/EU PPLAIs are indicated for the treatment only of schizophrenia [7, 8]. Better outcome might be expected in those with a diagnosis that reflects the bulk of research of a drug’s effects [9,10]. Continuation with treatment might also not be expected with all cases of some diagnoses, such as F29 and F31, which may not have a life-long course. It is also notable that other formulations of risperidone or paliperidone do have a formal license in the EU or USA for schizoaffective disorder (F25) [11,12] and bipolar disorder (F30/31) [13,14]. One recent observational study of LAIs looking at discontinuation rates over a year long period found no association between outcome and different diagnoses [15], but it is noteworthy that continuation rates in this study are much better than in similar observational studies of LAI treatment [16].

## Relapse

Patients prescribed antipsychotic polypharmacy were almost eight times more likely to experience relapse while on PP3M than those on monotherapy. Antipsychotic polypharmacy is highly controversial, but one recent study suggested that co-prescribing two antipsychotics was associated with a slightly lower risk of relapse than was monotherapy [17]. However, our results seem to show that concomitant antipsychotic treatment predicts higher risk of relapse while on PPLAIs. In this context, as noted above, the need for polypharmacy may indicate poor overall response to treatment and so higher rates of relapse might thus be anticipated.

All patients starting PP3M in this study were previously clinically stable on PP1M. Nonetheless, patients who have been previously considered for, or given, clozapine had a higher risk of relapse while on PPLAIs. So, despite clinical stability on starting PP3M the prior use or consideration of clozapine might well indicate treatment resistance at some level and therefore a greater risk of relapse (or at least poor outcome) when receiving nonclozapine antipsychotics.

As with discontinuation, patients with an F20 diagnosis were less likely to relapse than those with other diagnoses. Again, this is perhaps to be expected given the relative paucity of data relating to efficacy in non F20 diagnoses.

## Reasons for discontinuation

The most common reason for PP3M discontinuation was patient refusal. Almost half of patients switched back to PP1M after PP3M discontinuation. To some extent, this may indicate patients’ preference of PP1M over PP3M although our data did not indicate the stated reasons for the patients’ reasons for switching back to PP1M. Given that they were switched to the same drug, we can assume that the reason for the switch was not related to paliperidone itself, but rather the formulation and the frequency of its use. In conventional trials, PP1M and PP3M have near identical efficacy and tolerability [18].

## Comparison with other studies

Risperidone and paliperidone have demonstrated efficacy in Randomised Controlled Trials (RCTs) in the treatment of schizoaffective disorder [19,20] and are associated with a significant reduction in the risk of relapse [20,21]. Their use in bipolar disorder is supported by RCTs in which they are reported to delay the recurrence of mood episodes [22–24] and manic relapse [25,26]. Our study suggests that PP3M/PPLAI may be relatively less effective in these conditions than in schizophrenia. We are unaware of any other data which suggest this difference in outcomes. To the best of our knowledge, there are currently no other published observational studies looking at PP3M use in non F20 patients.

Our findings are consistent with other naturalistic studies affirming PP3M is highly effective in relapse prevention and reducing hospital admissions [27,28]. One recent study reports better outcomes for younger patients and suggests that earlier implementation of PP3M in therapy is critical for better long term outcomes and symptomatic remission [29]. Our findings on the effect of age on outcome are consistent with this study.

### Limitations

This was an open, naturalistic, and noninterventional analysis. As such there was no control group or blinding. By nature of being prescribed PP3M, the patients in this cohort were already stabilized on PP1M, so it might be expected that relapse and discontinuation rates would be low. Our dichotomous classification of ‘relapse’ may have been insensitive to some symptomatic worsening (although our definition was based on standard practice). The sample size was fairly small in respect to non-F20 diagnoses and participants were from one clinical environment—a socially deprived area of South East London—making it difficult to establish generalizability. Black people were over-represented in our sample which again may affect generalizability. We had no information on several factors that might influence outcome, such as substance or alcohol misuse. Lastly, diagnosis in practice is often less than certain and changes in diagnosis over time are far from unusual.

### What the study means for practice

Continuation with treatment at 2 years was more likely for F20-diagnosed patients and those of younger age. PPLAIs were less successful in other diagnoses but the outcomes were generally good, especially in respect to relapse. Patients previously considered for or given clozapine had a much higher likelihood of relapse and discontinuation from PPLAIs. We have demonstrated this association in several previous studies [30–33]. Patients co-prescribed two antipsychotics were at a greater risk of relapse and subsequent discontinuation than patients on PPLAI monotherapy. The best outcomes were seen in patients diagnosed with schizophrenia on PPLAI monotherapy who had never been prescribed or considered for clozapine.

## Data Availability

The data that support the findings of this study are available from david.taylor@slam.nhs.uk on request. Patient-identifying data will be removed to protect patient confidentiality.
